# Archetypal analysis of longitudinal visual fields for idiopathic intracranial hypertension patients presenting in a clinic setting

**DOI:** 10.1371/journal.pdig.0000240

**Published:** 2023-05-08

**Authors:** Joseph Branco, Tobias Elze, Jui-Kai Wang, Louis R. Pasquale, Mona K. Garvin, Randy Kardon, Mark J. Kupersmith

**Affiliations:** 1 New York Medical College, Valhalla, New York, United States of America; 2 Schepens Eye Research Institute, Harvard Medical School, Boston, Massachusetts, United States of America; 3 Department of Ophthalmology and Visual Sciences, University of Iowa, Iowa City, Iowa, United States of America; 4 Iowa City VA Center for the Prevention and Treatment of Visual Loss, Iowa City VA Healthcare System, Iowa City, Iowa, United States of America; 5 Department of Ophthalmology, Icahn School of Medicine at Mount Sinai, New York City, New York, United States of America; 6 Iowa City VA Center for the Prevention and Treatment of Visual Loss, Iowa City VA Healthcare System, Iowa City, Iowa, United States of America; 7 Department of Electrical and Computer Engineering, University of Iowa, Iowa City, Iowa, United States of America; Duke-NUS Medical School, SINGAPORE

## Abstract

We previously applied archetypal analysis (AA) using visual fields (VF) from the Idiopathic Intracranial Hypertension Treatment Trial (IIHTT) to derive a model, which quantified patterns (or archetypes [ATs] of VF loss), anticipated recovery, and identified residual VF deficits. We hypothesized that AA could produce similar results using IIH VFs collected in clinical practice. We applied AA to 803 VFs from 235 eyes with IIH from an outpatient neuro-ophthalmology clinic and created a clinic-derived model of ATs, with the relative weight (RW) and average total deviation (TD) for each AT. We also created a combined-derived model from an input dataset containing the clinic VFs and 2862 VFs from the IIHTT. We used both models to decompose clinic VF into ATs of varying percent weight (PW), correlated presentation AT PW with mean deviation (MD), and evaluated final visit VFs considered “normal” by MD ≥ -2.00 dB for residual abnormal ATs. The 14-AT clinic-derived and combined-derived models revealed similar patterns of VF loss previously identified in the IIHTT model. AT1 (a normal pattern) was most prevalent in both models (RW = 51.8% for clinic-derived; 35.4% for combined-derived). Presentation AT1 PW correlated with final visit MD (r = 0.82, p < 0.001 for the clinic-derived model; r = 0.59, p < 0.001 for the combined-derived model). Both models showed ATs with similar patterns of regional VF loss. The most common patterns of VF loss in “normal” final visit VFs using each model were clinic-derived AT2 (mild global depression with enlarged blind spot; 44/125 VFs; 34%) and combined-derived AT2 (near-normal; 93/149 VFs; 62%). AA provides quantitative values for IIH-related patterns of VF loss that can be used to monitor VF changes in a clinic setting. Presentation AT1 PW is associated with the degree of VF recovery. AA identifies residual VF deficits not otherwise indicated by MD.

## Author summary

Archetypal analysis is a type of machine learning that can extract common patterns from a dataset. In our work, we applied archetypal analysis to visual fields from eyes with papilledema due to idiopathic intracranial hypertension. We used archetypal analysis to create a model of the most common patterns of visual field loss and quantify the amount of representation of each pattern within the entire dataset. We then decomposed each visual field into a weighted linear combination of these patterns. We found that the weight of the pattern representing normal vision at presentation was associated with better visual field recovery. We also found that of 98 visual fields considered to be normal by the mean deviation, a commonly-used global measure of visual field loss, archetypal analysis detected a regional deficit in 58 (59%) of them. The mean deviation cannot account for minor or regional visual field deficits that archetypal analysis can detect. Therefore, archetypal analysis is a more powerful method to analyze visual field loss.

## Introduction

Idiopathic intracranial hypertension (IIH), a disorder that occurs most frequently in overweight women of childbearing age, causes papilledema, headache, diplopia, pulsatile tinnitus, and visual field (VF) loss [1]. Except for an enlarged blind spot, eyes with papilledema due to IIH display patterns of VF loss like those observed in eyes with glaucoma. Standardized automated perimetry, which tests threshold measurement of points in the central 30 degrees of the VF, is typically used to determine the severity and progression of disease. Until recently, expert consensus on the identification of patterns of VF deficits and global indices of visual function such as mean deviation (MD) and pattern standard deviation were virtually the only methods used to measure visual dysfunction in nonglaucomatous optic neuropathy. Regional VF defects that do not readily affect these global indices are reported as descriptive patterns of loss that are not quantified.

The Idiopathic Intracranial Hypertension Treatment Trial (IIHTT) characterized baseline and outcome VF features for study eyes presenting with mild VF loss and MD values between -7.00 and -2.00 dB [1–3]. At study outcome, while the MD recovered across the VF as a whole, the greatest improvements occurred in the periphery and in the region of the blind spot [3]. Though many eyes showed improvement in the VF over time with treatment, our prior report using machine learning analysis identified residual VF deficits at outcome in 97% of study eyes considered normal, having a MD of -2.00 dB or better [4].

Unsupervised machine learning has been used to quantify changes in VF deficits over time and monitor progression of VF loss in glaucoma, another disorder of the optic nerve [5–11]. We demonstrated that machine learning can track changes in VF loss patterns in the IIHTT [12]. However, machine learning analysis has not been used to assess IIH patients with a spectrum of VF loss outside the range encountered in the IIHTT, or for the evaluation of VFs collected from IIH patients in a clinical setting.

Archetypal analysis (AA), an unsupervised machine learning method, can quantify clinically recognizable patterns or archetypes (ATs) of VF loss for a particular optic nerve disorder by describing a VF as a sum of percent weights (PWs) of one or more ATs [13–15]. ^-^A disease-specific model can be used to measure PW changes in these ATs for each VF over time [7–11].

We studied whether AA of VFs from clinic IIH patients, with lesser and greater VF loss than patients in the IIHTT, would reveal new ATs not included in our previous IIHTT model [12]. We explored if the combination of clinic and IIHTT VFs would also reveal new ATs and how the relative weight (RW), a percent measure of the overall representation of each AT in the entire dataset, of previously identified patterns of VF loss [12] would change. We hypothesized that: 1) the clinic-derived AT patterns would show some differences from IIHTT-derived ATs [4], 2) clinic-derived AT PW at presentation would be associated with final visit MD for one or more ATs; and 3) AA would reveal residual VF defect patterns at final visit in eyes typically considered “normal” by MD.

## Methods

This study, which included the analysis of VFs from the IIHTT and Mount Sinai neuro-ophthalmology service. The study was approved by the Institutional Review Board (IRB) of the Icahn School of Medicine at Mount Sinai and is in compliance with ethical practices. The approval waived the requirement for individual consent as the patients were not available and the data collected were entirely de-identified. The study was conducted according to the principles expressed in the Declaration of Helsinki. The clinic dataset contained 803 VFs collected between 1997 and 2021 from both eyes of 118 patients, several of whom were treated a second time for recurrence of disease after more than one year of remission. Patients had clinical follow up including VF testing over the course of one year from presentation.

All patients had active papilledema and no optic atrophy by ophthalmoscopy at presentation. All patients met the modified Dandy criteria for IIH used in the IIHTT [1]. Patients were treated with a wide range of interventions depending on the clinical situation, including weight management alone, or in combination with acetazolamide, ventricular shunt, or optic nerve sheath fenestration. We used VFs from both eyes for all analyses of the clinic patients and IIHTT participants.

VF testing was performed using a Humphrey Field Analyzer with 24–2 SITA standard testing (Zeiss-Meditech Inc., Dublin CA). We only included VFs with fixation loss errors < 33%, and false-positive and false-negative errors < 15%. VF testing was performed once for both eyes at each visit: presentation, interim (any visit between presentation and final visit), and final (between five and twelve months after presentation). We analyzed VFs of 118 patients (235 eyes) at presentation, 58 patients (115 eyes) at an interim visit between one and 75 days, 57 patients (113 eyes) at an interim visit between 75 and 150 days, and 86 patients (170 eyes) at a final visit. Depending on MD, we characterized VF loss as severe (MD < -15 dB), moderate (-15 ≤ MD <-7 dB), or mild (-7 ≤ MD <-2 dB). We characterized VFs with MD ≥ -2 dB as normal by IIHTT criteria [2].

### Archetypal analysis

We used the open-source software package “archetypes” within the R statistical programming environment (R Development Core Team 2008) [15] to perform AA on all clinic VFs. The archetype function of the homonymous R library was called with default values for maxIterations (100), minImprovement (square root of machine precision eps), and maxKappa (1000), without specifying weights to the archetypes. We used the total deviation (TD) values at each VF location tested, extracted from these VFs, to generate ATs. AA generated a model of ATs, representative of major patterns of VF loss, along with the average TD and RW of each AT. We used ten-fold cross validation to select the number of ATs wherein the data were divided into 10 even subsets, each patient was used once for the testing set, and the other nine were used as the training set for our models. No patients appeared in more than one subset. We calculated the residual sum of squared errors (RSS) using two to 20 ATs and plotted it against the number of ATs. The region where the RSS curve began to flatten was used to determine whether our initial choice of number of ATs to be used in our model were reasonable (S1 Fig). As the RSS curve flattened in the region that included 14 ATs (like our IIHTT model), we chose a 14-AT model to characterize the clinic dataset. We repeated this process to create a second model (combined-derived) using a data set containing clinic (803 VFs) and IIHTT (2862 VFs) data (3665 VFs in total).

Using the clinic-derived and combined-derived models individually, we performed separate decompositions of the clinic VFs into ATs. Decomposition resolved each VF into a sum of PWs of one or more ATs, with all PWs summing to 100%. As explained in our previous studies, we deemed an AT PW of ≥ 9% as clinically meaningful to avoid potential clinically insignificant variability [12]. As a comparison we also decomposed the VFs using a third model, the IIHTT-derived model, previously created from the IIHTT VFs alone [12].

### Association for AT PWs and MD

We evaluated whether the PW for one or more ATs was associated with the MD at presentation or final visit. Based on the prior report showing an association between presentation AT1 (a normal VF pattern) PW and MD using VFs from the IIHTT [4], we evaluated if presentation clinic-derived or combined-derived AT1 PW could predict the extent of recovery at final visit. For this evaluation, we calculated the mean presentation AT1 PW using presentation VFs for clinic patients with final visit VFs. We divided patients into two groups, one with presentation AT1 PWs above the mean and the other with presentation AT1 PWs below the mean. We then plotted the average MD over time for each group. While we previously used one standard deviation above the mean presentation AT1 PW to divide patients into two groups [12], due to fewer subjects in the clinic dataset, we used the mean as the divider to allow for a similar number of patients in each group.

### Statistical analyses

We performed all statistical analyses using MedCalc and the open-source Python packages NumPy, SciPy, and Matplotlib. We set the significance level to α = 0.05. We used non-parametric t-tests to compare the mean age and mean MD between clinic patients and IIHTT subjects. We used chi-square tests to compare the percentage of women and percentage of patients with different severities of VF loss between clinic patients and IIHTT subjects. We used Spearman’s method to correlate AT1 PW with MD. We used the Mann Whitney U Test to evaluate for any significant difference in average MD and mean AT PW values between patients with presentation AT1 PWs above and below the mean.

## Results

### Demographics and distribution of VF loss among clinic patients

The mean age was 32 ± 11 years (range 10–61) for clinic patients and 29 ± 8.4 years (range 18–55) for IIHTT patients (p = 0.021). There was a smaller percentage of women in the clinic dataset (105/118, 89.0%) than in the IIHTT dataset (161/165, 97.6%; p < 0.001).

The average MD at presentation of clinic eyes was worse than for all eyes enrolled in the IIHTT (-4.2 ± 6.2 dB vs.-2.9 ± 1.3 dB; p < 0.001). The clinic dataset included cases of moderate and severe VF loss not included in the IIHTT dataset (Table 1). The clinic patient VFs were significantly different from the IIHTT VFs for all degrees of severity.

**Table 1:** Comparison of clinic and Idiopathic Intracranial Hypertension Treatment Trial (IIHTT) eyes using mean deviation (MD) characteristics (both study and fellow eyes were included). *The range of MD values for study eyes included in the IIHTT was -7.00 to -2.00 dB, but fellow eyes were typically better than the study eye.

	Presentation			Final Visit		
	Clinic (n = 235)	IIHTT (n = 330)	P-value	Clinic (n = 170)	IIHTT (n = 248)	P-value
average MD (dB)	-4.2 ± 6.2	-2.9 ± 1.3	<0.001	-3.1 ± 5.4	-2.0 ± 2.0	<0.001
quartile 1 MD (dB)	-4.9	-3.7	-	-3.1	-2.7	-
quartile 2 MD (dB)	-2.5	-2.8	-	-1.7	-1.6	-
quartile 3 MD (dB)	-1.0	-2.1	-	-0.4	-0.9	-
quartile 4 MD (dB)	2.2	0.9	-	4.0	1.3	-
MD IQR (dB)	3.9	1.6	-	2.7	1.8	-
# eyes with MD <-15 dB	13 (6%)	0 (0%)	<0.001	6 (4%)	1 (<1%)	<0.001
# eyes with -15≤ MD <-7 dB	18 (8%)	0 (0%)	<0.001	14 (8%)	3 (1%)	<0.001
# eyes with -7 ≤ MD <-2 dB	100 (43%)	261 (79%)	<0.001	52 (31%)	95 (38%)	0.14
# eyes with MD ≥-2 dB	104 (44%)	69 (21%)*	<0.001	98 (58%)	149 (60%)	0.68

### Archetypal analysis models

**Fig 1:**
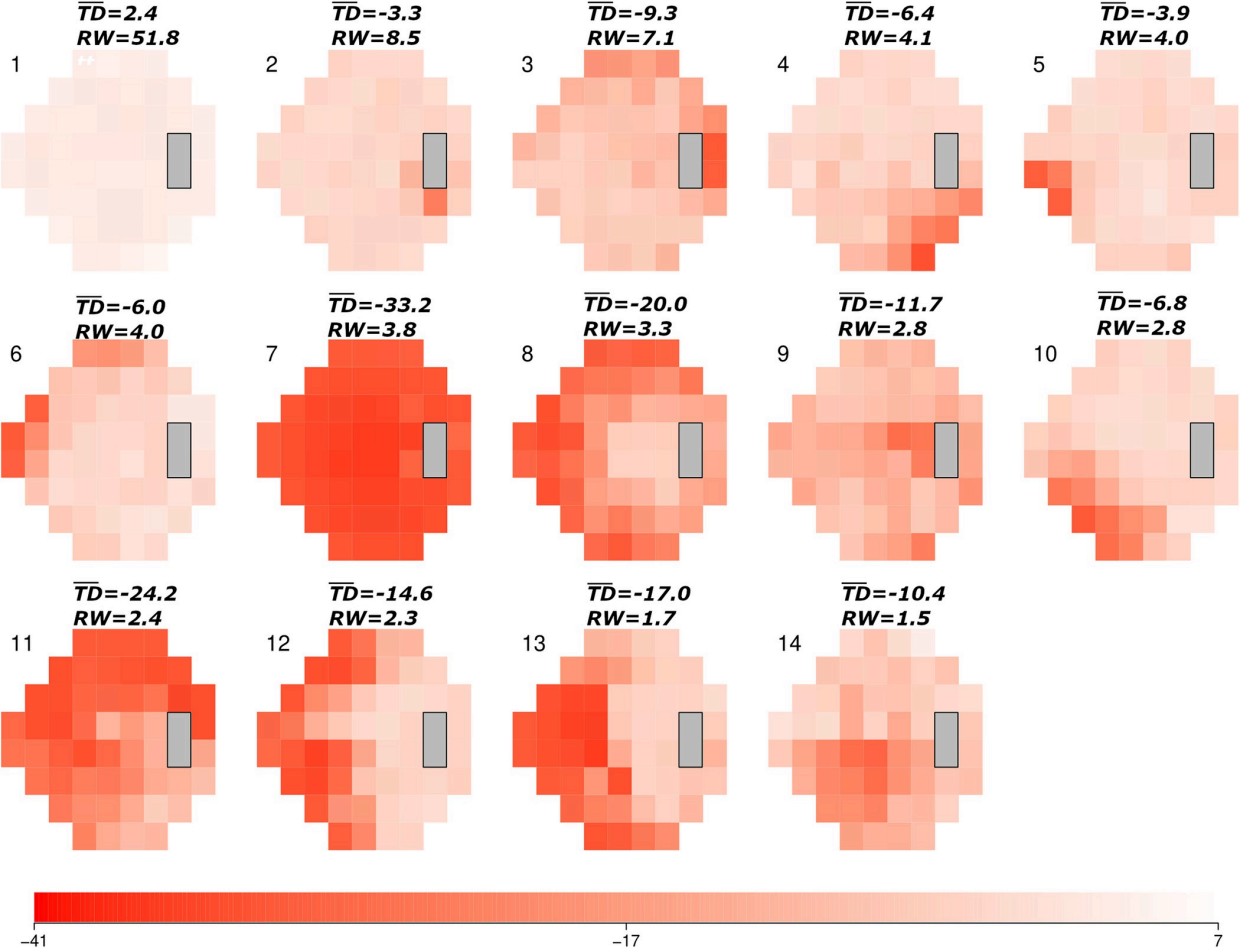
14-archetype (AT) model derived from the neuro-ophthalmology clinic dataset. ATs are shown in descending order of relative weight (RW; expressed as a percent), representing their amount of representation within the dataset. The scale (bottom) denotes average total deviation ($\bar{TD}$) values (range -41 to 7 dB). Each AT pattern is shown with its corresponding $\bar{TD}$ and RW value.

The clinic-derived AT model showed the range of clinically recognizable IIH patterns ATs, each with an associated average TD and RW value (Fig 1). The three major clinic-derived ATs were AT1 (a normal VF pattern; RW = 51.8%; average TD = 2.4 dB), AT2 (a mild global depression with enlarged blind spot; RW = 8.5%, average TD = -3.3 dB), and AT3 (a mild global depression pattern with enlarged blind spot and partial superior arcuate defect; RW = 7.1%, average TD = -9.3 dB).

The combined-derived AT model also showed the range of clinically recognizable patterns IIH ATs each with an associated average TD and RW value (S2 Fig). The three major combined-derived ATs were AT1 (a normal VF pattern; RW = 35.4%; average TD 2.2 dB), AT2 (a near-normal VF pattern; RW = 17.8%; average TD = 0.8 dB), and AT3 (a mild global depression pattern with superior arcuate defect). Although AT patterns were similar between both models, the average TD and RW values differed as the combined model included the IIHTT VFs.

### Comparison of archetypes for each IIH model

The average number of abnormal clinic-derived ATs of meaningful PW for each presentation VF from the clinic dataset was 1.7 ± 1.2. Of 235 presentation VFs, 201 (86%) VFs were decomposed into at least one but no more than five abnormal clinic-derived ATs (any AT other than AT1) of meaningful PW. The remaining 34 VFs (14%) had no abnormal clinic-derived AT of meaningful PW. AT1 was the dominant AT in all 34 VFs with no clinic-derived AT of meaningful PW and 32/34 (94%) had a MD ≥ -2.00 dB. Clinic-derived AT2 was the most common abnormal AT, occurring in 96 (41%) presentation VFs. Clinic-derived AT3 (81 VFs; 34%), AT5 (inferior nasal wedge defect; 40 VFs; 17%), and AT6 (superior nasal wedge defect with partial superior arcuate defect; 40 VFs; 17%; Fig 1), were the next most common ATs.

The average number of abnormal combined-derived ATs of meaningful PW for each presentation VF from the clinic dataset was 2.0 ± 1.1. Of 235 presentation VFs, 217 (92%) VFs were decomposed into at least one but no more than five abnormal combined-derived ATs. The remaining 18 VFs (8%) had no abnormal clinic-derived AT of meaningful PW. AT1 was the dominant AT in all 18 VFs with no clinic-derived AT of meaningful PW and 17/18 (94%) had a MD ≥ -2.00 dB. Combined-derived AT2 was the most common abnormal AT of meaningful PW, occurring in 122 (52%) presentation VFs. Combined-derived AT3 (62 VFs; 26%) and AT7 (superior-nasal defect; 52, VFs; 22%; S2 Fig), were the next most common ATs of meaningful PW. The clinic-derived model showed AT patterns similar to those in the IIHTT-derived model. The combined-derived model showed AT patterns similar to those in the clinic-derived model (S1 Table and S2 Table).

### AT PWs association with MD (Table 2)

**Fig 2:**
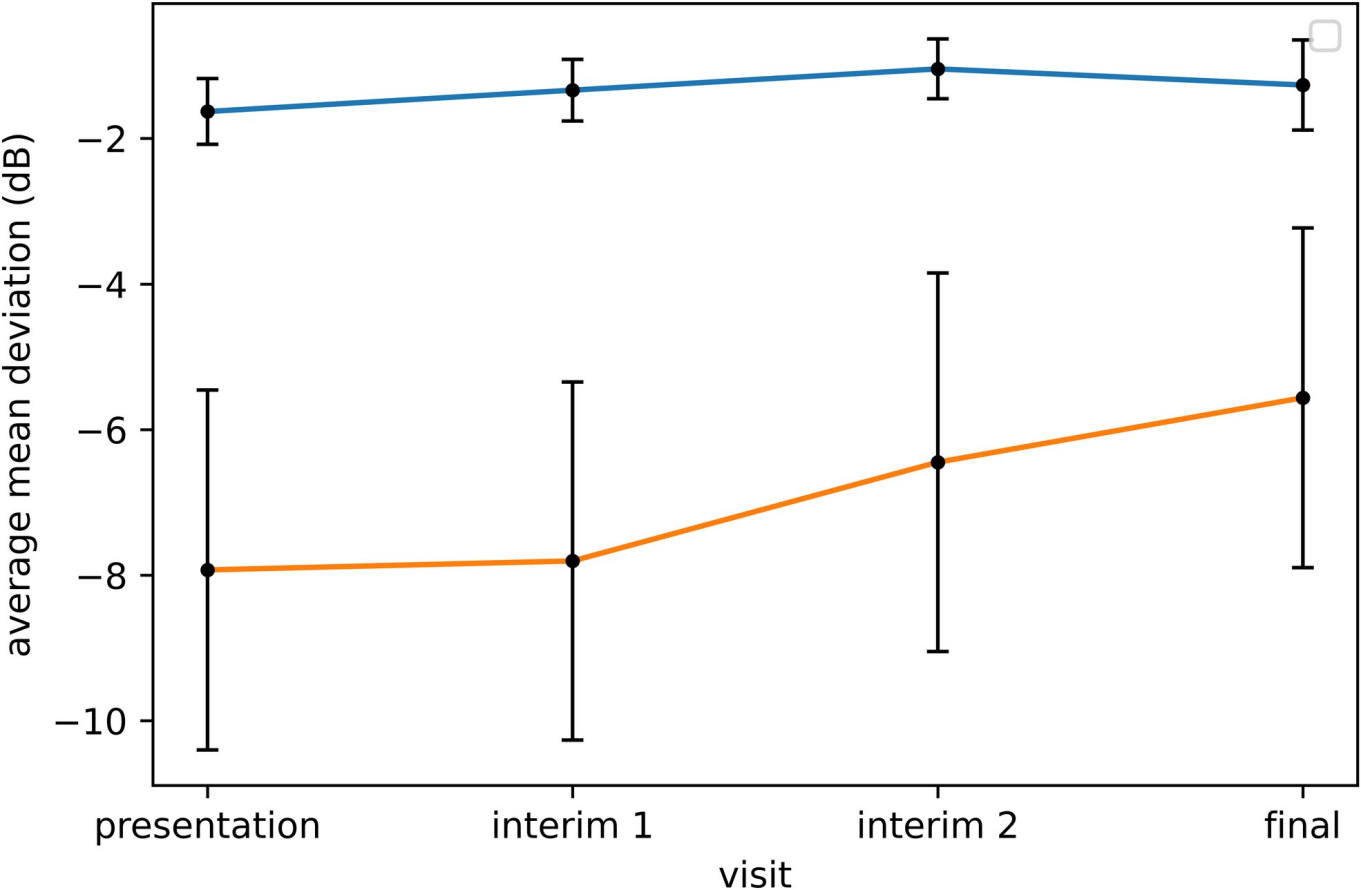
Average mean deviation (dB) over time for eyes with presentation clinic-derived archetype-1 (AT1) percent weight (PWs) above (blue line) and below (orange line) the mean, showing 95% confidence intervals at each time point.

The clinic-derived AT1 PW correlated with MD for VFs collected at presentation (r = 0.80, p < 0.001) and final visits (r = 0.82, p < 0.001). The average MD values at presentation and final visits were significantly higher for VFs with presentation clinic-derived AT1 PWs above rather than below the mean (Fig 2). The mean clinic-derived AT1 PW at final visit remained significantly higher (p < 0.001) for eyes with presentation clinic-derived AT1 PWs above (72%; 95% CI: 67–76%) rather than below (44%; 95% CI: 35–54%) the mean.

**Table 2:** The mean presentation archetype percent weights (AT PWs) and average mean deviation (MD) values at presentation and final visits for clinic visual fields (VFs) with presentation AT PWs above and below the mean. 95% confidence intervals are shown in parentheses.

	Presentation				Final Visits		
	Mean AT PW (%)	Average MD for VFs with presentation AT PWs below the mean (dB)	Average MD for VFs with presentation AT PWs above the mean (dB)	p-value	Average MD for VFs with presentation AT PWs below the mean (dB)	Average MD for VFs with presentation AT PWs above the mean (dB)	p-value
Clinic-derived AT1	47 (43–51)	-7.92 (-10.40, -5.46)	-1.63 (-2.08, -1.18)	< 0.001	-5.56 (-7.89, -3.23)	-1.27 dB (-1.89, -0.64)	< 0.001
Combined-derived AT1	34 (30–38)	-6.16 (-7.75, -3.44)	-1.47 (-2.06, -0.88)	< 0.001	-3.82 (-5.41, -2.21)	-1.04 (-1.59, -0.50)	< 0.001

The combined-derived AT1 PW correlated with MD for VFs collected at presentation (r = 0.57, p < 0.001) and final visit (r = 0.59, p < 0.001). The average MD values at presentation and final visits were significantly higher for VFs with presentation combined-derived AT1 PWs above rather than below the mean. The mean combined-derived AT1 PW at final visit remained higher (p = 0.002) for VFs with presentation combined-derived AT1 PWs above (53%; 95% CI: 47–60%) rather than below (35%; 95% CI: 27–43%) the mean.

### Residual Defects in Final Visit VFs

Of the 170 final visit VFs, 125 (74%) were decomposed into at least one abnormal clinic-derived AT of meaningful PW. The most common abnormal ATs were AT2 (44 VFs; [35%]) and AT3 (43 VFs [34%]). One hundred forty-nine (88%) final visit VFs (170) were decomposed into at least one abnormal combined-derived AT of meaningful PW. The most common abnormal ATs were AT2 (93 VFs; [62%]) and AT3 (37 VFs; [25%]).

Of the 98/170 (58%) VFs considered “normal” by MD ≥ -2.00 dB at final visit, 58/98 (59%) were decomposed into at least one abnormal clinic-derived AT of meaningful PW. The most common abnormal ATs were AT2 (23 VFs; [40%]) and AT3 (22 VFs; [38%]). Using combined-derived ATs, 80/98 (82%) “normal” final visit VFs were decomposed into at least one abnormal combined-derived AT of meaningful PW. The most common abnormal ATs were AT2 (63 VFs [78%]) and AT4 (an enlarged blind spot pattern; 14 VFs [18%]).

### Case examples for decomposition of VFs using clinic-derived, IIHTT-derived, and combined ATs

**Fig 3:**
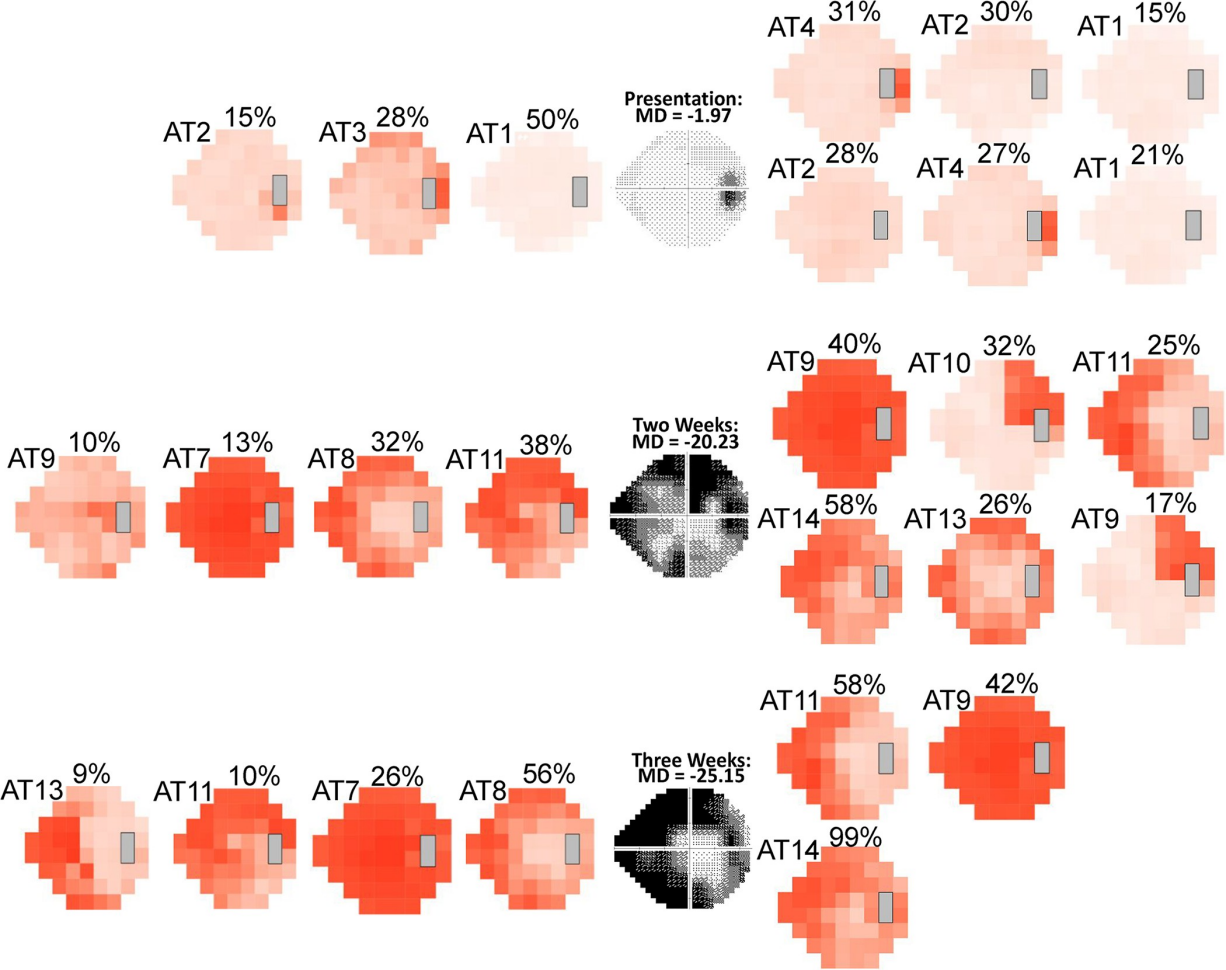
Case example of archetype (AT) decomposition of visual fields (VFs) from an eye with mild loss at presentation and worsening of loss at two and three weeks (top to bottom). Decompositions are shown using the clinic-derived model (left of grayscale VF), combined-derived model (upper right of grayscale VF) and Idiopathic Intracranial Hypertension Treatment Trial (IIHTT)-derived model (lower right of grayscale VF). All three models identify an enlarged blind spot at presentation. Only the clinic-derived and combined-derived AT models identify a pattern of global loss beginning at two weeks with worsening at three weeks.

**Fig 4:**
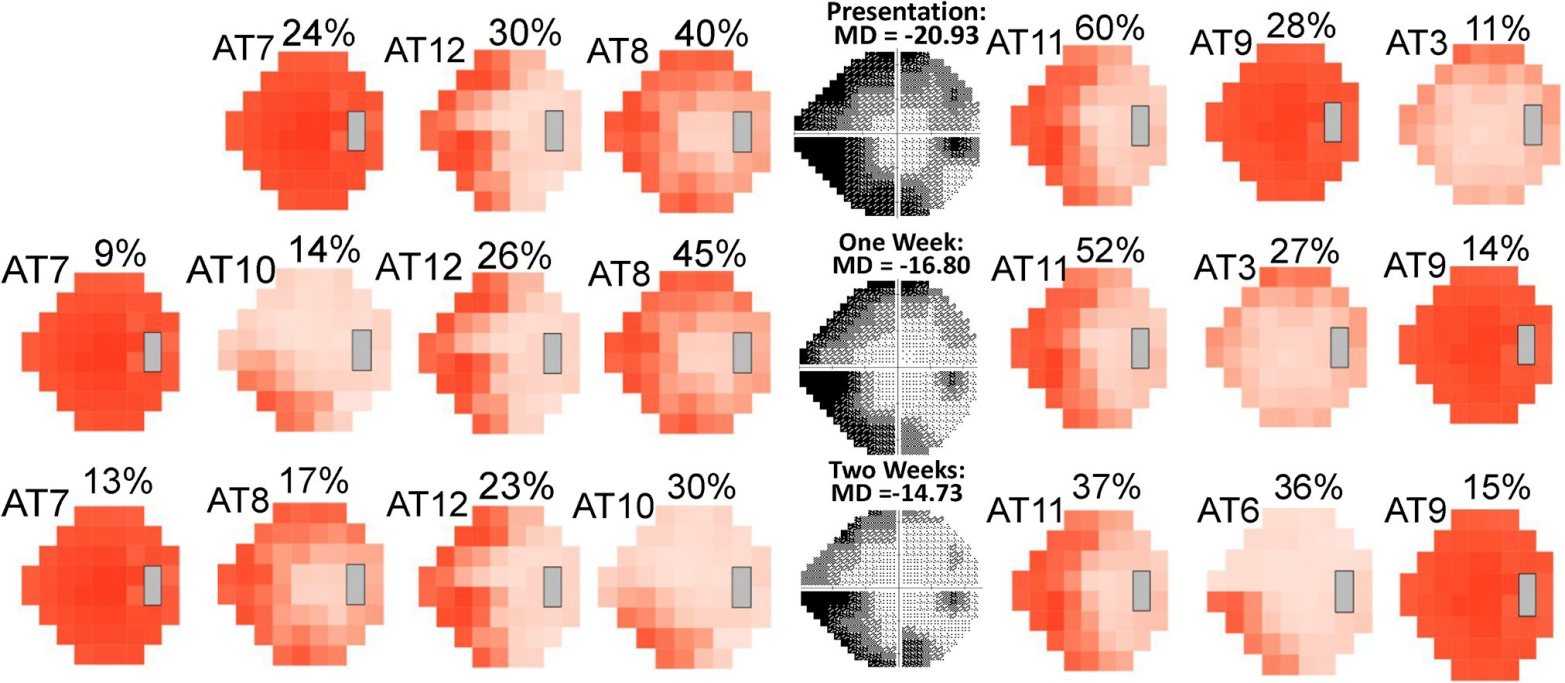
Case example of archetype (AT) decomposition of visual fields (VFs) from an eye with severe loss at presentation and change over time. Decompositions are shown using the clinic-derived model (left of grayscale VF) and combined-derived model (right of grayscale VF). Both models show similar patterns.

**Fig 5:**
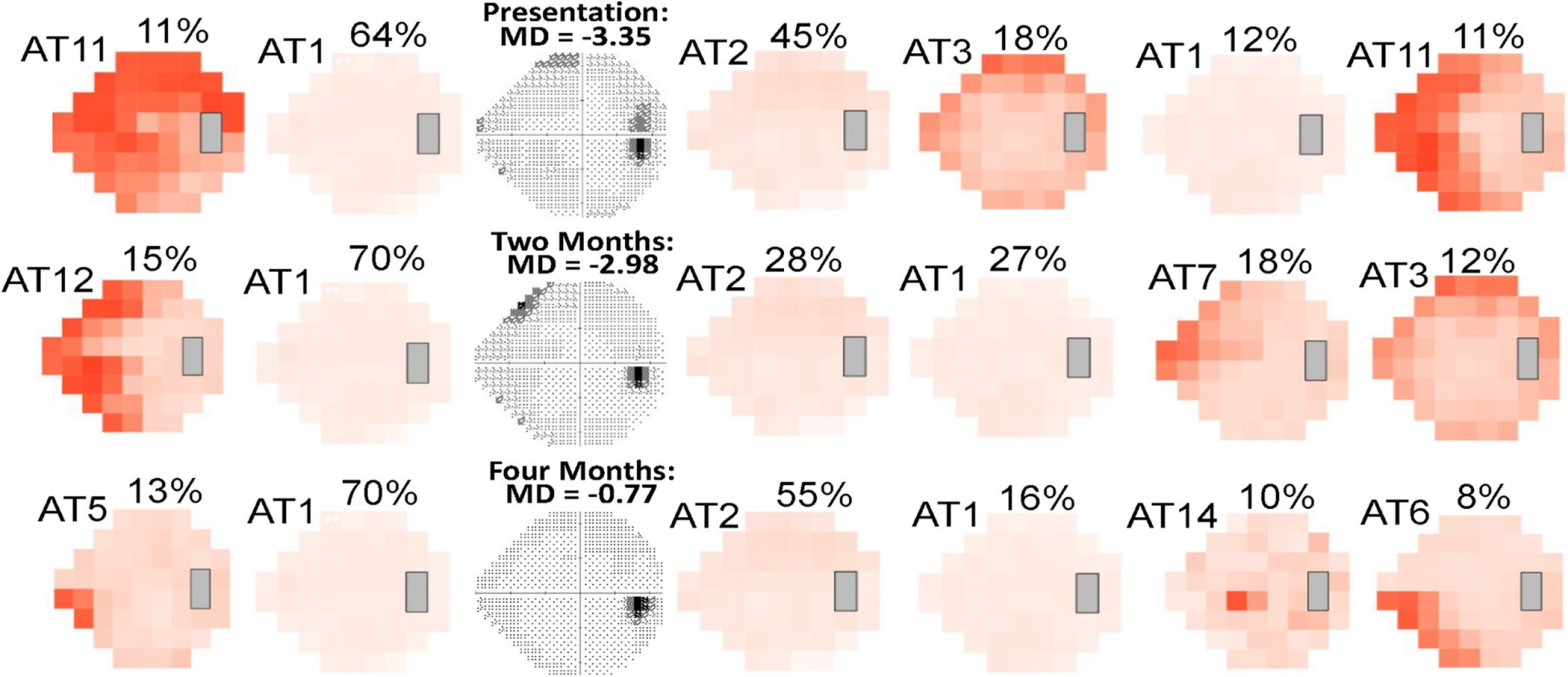
Case example of archetype (AT) decomposition of VFs from an eye with mild loss at presentation and residual deficits despite a mean deviation (MD) ≥ -2.00 dB at final visit. Decompositions are shown using the clinic-derived model (left of grayscale visual field [VF]) and combined-derived model (right of grayscale VF). The combined-derived model also revealed an inferior nasal deficit, but with a percent weight (PW) of 8% (below the cutoff to be considered meaningful).

Depending on which of the three IIH AT models we used, different patterns of disease progression emerged. In Fig 3, an eye with mild VF loss at presentation progressed to severe loss at three weeks. At two and three weeks, the clinic-derived and combined-derived models, but not the IIHTT-derived model show a component of severe global loss (see discussion). The VF shown in Fig 4 had severe loss at presentation and had central VF improvement at two weeks. In this case, both the clinic-derived and combined-derived models show a similar amount of recovery and VF patterns at two weeks. In Fig 5, the VF showed mild loss at presentation and improved over time. Despite a normal MD at four months, both models identify a residual inferior nasal deficit, although the combined-derived AT6 PW is just below the 9% cutoff, which is considered meaningful.

## Discussion

AA identified quantifiable and IIH-specific patterns of VF loss in VFs collected in a neuro-ophthalmology clinic from IIH patients with a wide spectrum of VF loss, from minimal to severe, as determined by MD. We combined clinic and IIHTT VFs to derive a new IIH AT model with ATs that more completely characterized the patterns of VF loss that can occur with this disorder. We identified an association between presentation AT1 PW and final visit MD using both the clinic-derived and combined-derived models. AA revealed specific regional deficits in VFs at presentation, and residual deficits at final visit in VFs from eyes considered “normal” based on a MD ≥ -2.00 dB.

Our previous report utilized AA to investigate VF data from the IIHTT, in which enrolled patients had a VF MD between -2.00 dB and -7.00 dB in the study eye [1]. However, many patients with IIH have VF loss outside the restricted range for inclusion in the IIHTT. Our results expand on our prior use of AA in the characterization of IIH-related VF loss by applying it to VFs collected in a less-controlled clinic setting from patients with a wider range of VF severity, including cases without obvious, as well as moderate and severe VF loss.

We identified numerous AT patterns of VF loss in the clinic and combined datasets similar to those identified in our prior study on IIHTT VFs [12]. These patterns included a normal VF, enlarged blind spot, inferior temporal wedge, superior nasal defect, inferior nasal defect, and inferior altitudinal defect. We identified new patterns in the clinic-derived ATs that represented more severe loss, including clinic-derived AT7 (a pattern of severe global loss) and clinic-derived AT8 (a severe partial peripheral defect), neither of which was included in the IIHTT-derived AT set. When clinic and IIHTT data were combined, both AT patterns re-emerged as combined-derived AT9 (severe global loss pattern) and combined-derived AT11 (severe partial peripheral rim defect). Most importantly, each set of visual fields produced patterns of VF loss that occur with IIH. Since the IIHTT did not include cases of moderate or severe VF loss, the inclusion of cases with presentation MDs worse than -7.00 dB expanded the total number of ATs that describe IIH-related VF loss. This is not surprising given the wider range and overall increased severity of VF loss in the clinic dataset relative to the IIHTT dataset. Our inclusion of eyes with MD > -2.00 dB at presentation is reflected by the greater RW and average of clinic-derived and combined-derived AT1 relative to IIHTT-derived AT1 (see supplement data; RW 30.9%; average TD -2.29 dB). The case in Fig 3 highlights, the importance of complete range of VF loss for an IIH AT model. As the IIHTT AT model alone did not have a severe global loss AT [4], this model did not show an AT with the most severe loss.

The presentation AT1 PW obtained from decomposition of VFs using both the clinic-derived and combined-derived models correlated with the final visit MD. This illustrates that AA has the potential to predict an outcome based on the VF at presentation. For both AT models, the change in AT1 PW over time closely followed the trend in MD. This reinforces that AT1 PW appears to be a reasonable surrogate for MD. AT1 can be used to quantify the amount of normal character in a VF, in addition to possibly predicting recovery and identifying patients more likely to benefit from intensive treatment. We previously noted more than one abnormal AT PW at baseline was associated with MD at outcome for the IIHTT VFs [4]. However, in this study, we could not evaluate if other presentation ATs were predictive of the final visit MD in clinic patients for two reasons. First, no other AT PWs significantly correlated with MD at presentation and final visits. Second, no other AT had a large enough RW to establish a mean presentation PW that divided eyes with AT PWs below or above the mean into groups of similar sizes.

The case examples show AA decomposition of IIH VFs adds detail to the assessment of changes in VFs over time to complement the MD. The MD is derived from thresholds at points in the field considered most reliable for data capture. Thus, values from tested points in the peripheral VF have limited contribution to the MD. Our case example in Fig 3 also highlights the need to have an AT model that encompasses all degrees of VF loss, as the severe global loss AT seen in both the clinic-derived and combined-derived models did not occur in the IIHTT-derived model. Therefore, the inclusion of more severe clinic cases effectively expands the original model.

AA of final VFs, which were collected months after treatments were initiated, revealed residual deficits for both models that are not obvious in VFs with normal or near-normal MD. AA decomposition revealed residual deficits in 59% of final visit VFs with MD ≥ -2.00 dB using the clinic-derived model, and 82% using the combined-derived model. Previously, we identified residual deficits in 97% of study eyes included in the IIHTT [4]. The difference in this current study is likely due to the inclusion of both eyes from each patient (rather than only from the worse eye) and eyes with presentation MD values ≥ -2.00 dB.

There were limitations and differences in this study compared with the IIHTT. The number of patients included in this study was smaller and the age range was wider. Since our data were derived from patients not treated in a controlled trial, visits did not occur at standardized time points. Some patients had final visits after six months (the endpoint of the IIHTT) or did not return for re-evaluation. We used both eyes from each patient in our analysis, whereas in our previous study, we used only the IIHTT study eyes (with worse MD) [12]. As noted above, we included eyes with MD ≤ -7.00 dB and > -2.00 dB at presentation.

Future work will explore the association between regional residual VF deficits detected by AA and structural changes to the optic nerve detected by optical coherence tomography after resolution of optic disc edema at later time points. We suggest that regional VF deficits may correlate with corresponding areas of structural damage to the optic nerve. An approach that combines AA of VFs and imaging may provide a better understanding or method to evaluate and monitor IIH.

## Supporting information

S1 Fig Residuals sum of squares (RSS) plot created for selection of clinic-derived archetype (AT) model. Flattening of the slope near 14 ATs supported the use of a 14-AT model.
(PDF)

S2 FigThese are the 14 archetypes (ATs) derived from the combined neuro-ophthalmology practice and Idiopathic Intracranial Hypertension Treatment Trial (IIHTT) datasets.ATs are shown in descending order of relative weight (RW), representing their frequency within the dataset. The scale (bottom) denotes average total deviation ($\bar{TD}$) values (range -41 to 7 dB). Each AT pattern is shown with its corresponding $\bar{TD}$ and RW value.(PNG)

S1 Table Relative weights (RW) and average total deviation (TD) values for clinic-derived archetypes (ATs) and corresponding IIHTT-derived archetypes of similar patterns. (DOCX)

S2 Table Relative weights (RW) and average total deviation (TD) values for combined-derived archetypes (ATs) and corresponding clinic-derived archetypes of similar patterns. (DOCX)

S1 Data Total deviation (TD) values of each visual field test point and the overall mean deviation (MD) of each visual field from clinic patient eyes. Columns H through BG correspond to the 54 points tested in a 24–2 visual field. Points 26 and 35 correspond to the physiologic blind spot and were omitted.
(XLSX)
