# Supplementary figures and images for: Archetypal analysis of longitudinal visual fields for idiopathic intracranial hypertension patients presenting in a clinic setting
Source: PLOS Digit Health. 2023 May 8;2(5):e0000240. doi: 10.1371/journal.pdig.0000240 (PMC10166546; doi:10.1371/journal.pdig.0000240)

## Residual Sums of Squares

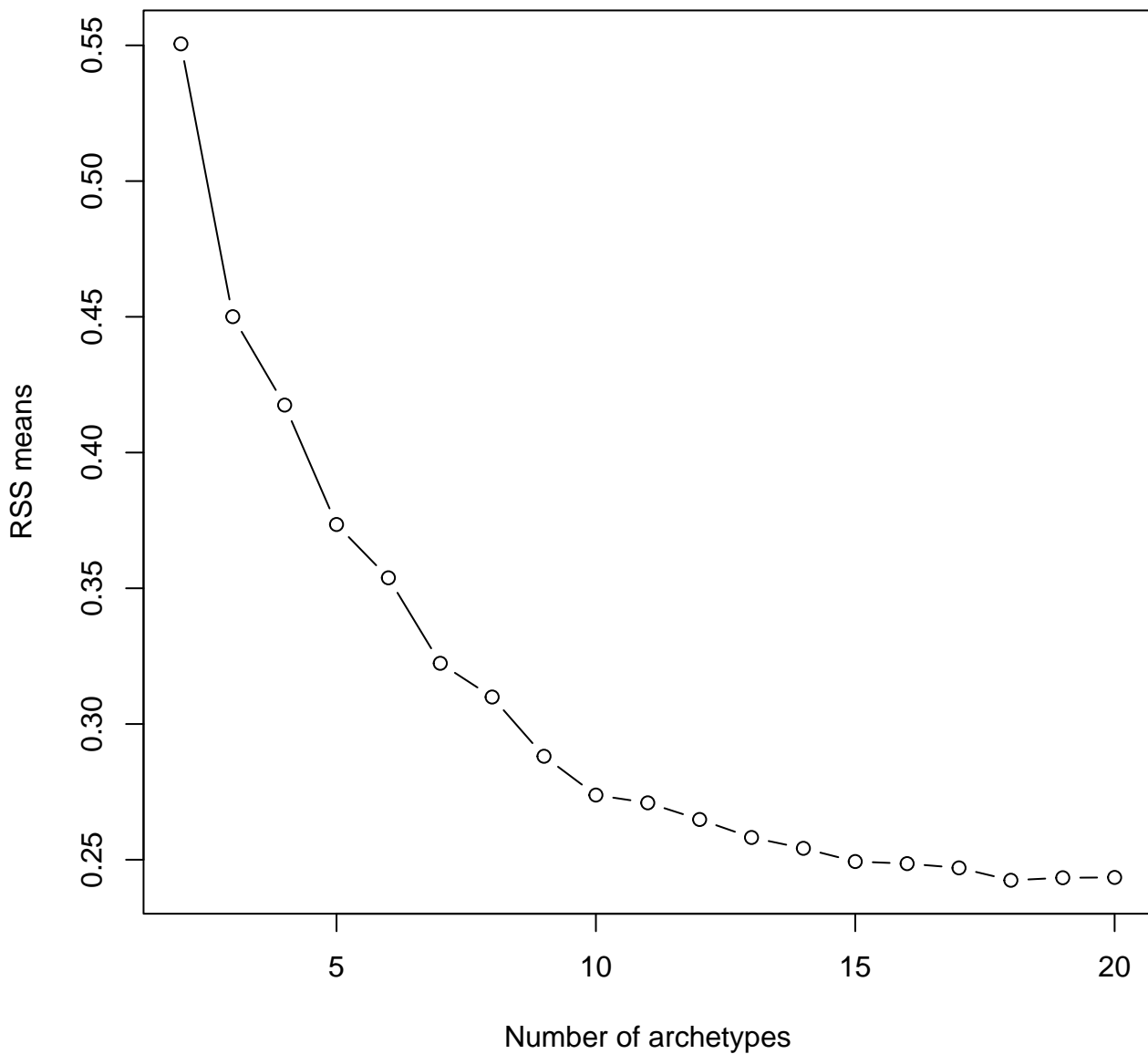

Supplement: S1 Fig — Flattening of the slope near 14 ATs supported the use of a 14-AT model. (PDF) [file pdig.0000240.s001.pdf]

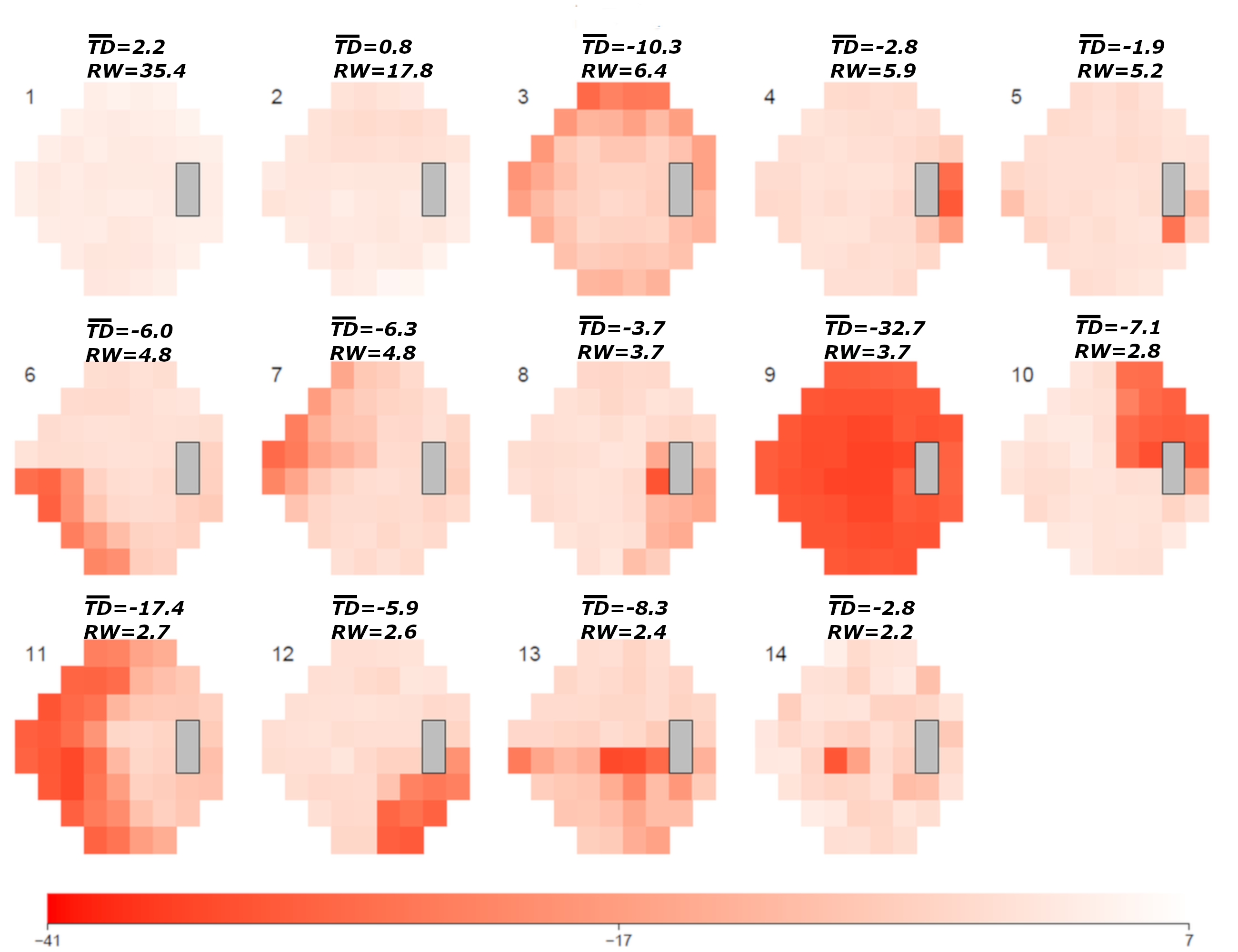

Supplement: S2 Fig — ATs are shown in descending order of relative weight (RW), representing their frequency within the dataset. The scale (bottom) denotes average total deviation (TD¯) values (range -41 to 7 dB). Each AT pattern is shown with its corresponding TD¯ and RW value. (PNG) [file pdig.0000240.s002.png]
