# Supplementary material for: Archetypal analysis of longitudinal visual fields for idiopathic intracranial hypertension patients presenting in a clinic setting
Source: PLOS Digit Health. 2023 May 8;2(5):e0000240. doi: 10.1371/journal.pdig.0000240 (PMC10166546; doi:10.1371/journal.pdig.0000240)
Supplement: S1 Table — (DOCX) [file pdig.0000240.s003.docx]

| Clinic-derived AT | RW (%) | Avg. TD (dB) | IIHTT-derived AT | RW (%) | Avg. TD (dB) |
| --- | --- | --- | --- | --- | --- |
| AT1 | 51.8 | 2.44 | AT1 | 30.9 | -2.29 |
| AT2 | 8.45 | -3.27 | AT7 | 4.59 | -2.57 |
| AT4 | 4.13 | -6.41 | AT12 | 2.64 | -4.39 |
| AT6 | 4.01 | -5.94 | AT6 | 4.97 | -6.56 |
| AT8 | 3.28 | -19.9 | AT13 | 2.14 | -18.4 |
| AT10 | 2.77 | -6.82 | AT5 | 5.18 | -5.90 |
| AT11 | 2.35 | -24.2 | AT14 | 1.87 | -21.0 |
| AT14 | 1.47 | -10.4 | AT10 | 2.94 | -7.79 |

S1 Table: Relative weights (RW) and average total deviation (TD) values for clinic-derived archetypes (ATs) and corresponding IIHTT-derived archetypes of similar patterns.
