# Supplementary material for: Archetypal analysis of longitudinal visual fields for idiopathic intracranial hypertension patients presenting in a clinic setting
Source: PLOS Digit Health. 2023 May 8;2(5):e0000240. doi: 10.1371/journal.pdig.0000240 (PMC10166546; doi:10.1371/journal.pdig.0000240)
Supplement: S2 Table — (DOCX) [file pdig.0000240.s004.docx]

| Combined-derived AT | RW (%) | Avg. TD (dB) | Clinic-derived AT | RW (%) | Avg. TD (dB) |
| --- | --- | --- | --- | --- | --- |
| AT1 | 35.4 | 2.16 | AT1 | 51.8 | 2.44 |
| AT2 | 17.8 | 0.82 | AT1 | 51.8 | 2.44 |
| AT4 | 5.89 | -2.83 | AT2 | 8.45 | -3.27 |
| AT5 | 5.15 | -1.87 | AT2 | 8.45 | -3.27 |
| AT6 | 4.82 | -5.96 | AT10 | 2.77 | -6.82 |
| AT7 | 4.79 | -6.26 | AT6 | 4.01 | -5.94 |
| AT9 | 3.66 | -32.7 | AT7 | 3.81 | -33.2 |
| AT11 | 2.65 | -17.4 | AT8 | 3.28 | -19.9 |
| AT13 | 2.39 | -8.26 | AT14 | 1.47 | -10.37 |

S2 Table: Relative weights (RW) and average total deviation (TD) values for combined-derived archetypes (ATs) and corresponding clinic-derived archetypes of similar patterns.
